# Massive hemoptysis as the initial presentation of Behçet disease complicated by multisite thromboembolism: A case report

**DOI:** 10.1097/MD.0000000000049496

**Published:** 2026-06-26

**Authors:** Mohammad Obada Alsadi, Amr Hodaifa, Areej Alboudy, Wajd Tareq, Reem Akasha, Bashar Quteishat, Mushrega Abdalla

**Affiliations:** a Faculty of Medicine, Damascus University, Damascus, Syrian Arab Republic; b Respiratory System Diseases Department, Faculty of Medicine, Damascus University, Damascus, Syrian Arab Republic; c Internal Medicine Department, Faculty of Medicine, Damascus University, Damascus, Syrian Arab Republic; d Yarmouk University, Irbid, Jordan; e University of Gezira, Sudan; f Al-Essra Hospital, Amman, Jordan; g University of Khartoum, Khartoum, Sudan.

**Keywords:** Behçet disease, hemoptysis, intracardiac, thromboembolism

## Abstract

**Rationale:**

Behçet disease (BD) is a systemic vasculitis with highly variable presentations. While vascular involvement is a major complication and a leading cause of mortality, the pattern can vary dramatically. This report details a severe and rare manifestation dominated by extensive thrombotic events.

**Patient concerns:**

A 20-year-old male with a history of recurrent oral ulcers presented with massive hemoptysis and a persistent fever of 39°C. Laboratory findings revealed severe anemia and a positive pathergy test.

**Diagnoses:**

Imaging revealed extensive thrombosis involving the inferior vena cava, right atrium, multiple pulmonary veins, and an intracardiac thrombus in the right ventricle. The patient met the International Criteria for Behçet’s Disease criteria with 4 points, confirming a diagnosis of BD complicated by severe multisite thromboembolism.

**Interventions:**

The patient was managed using a carefully timed, sequential approach. Treatment included initial measures for immediate hemostasis, followed by aggressive immunosuppression with high-dose corticosteroids, and subsequent tailored anticoagulation and biological immunotherapy.

**Outcomes:**

The therapeutic approach resulted in significant clinical and radiological improvement, successfully resolving the life-threatening thrombi without inducing recurrent hemorrhage.

**Lessons:**

BD can manifest with an aggressive, purely thrombotic phenotype involving multisite venous and intracardiac structures without classic arterial aneurysms. In cases presenting with concurrent massive hemoptysis, a sequential strategy – prioritizing immediate hemostasis before aggressive immunosuppression and cautious anticoagulation – is crucial for safe and effective management.

## 1. Introduction

As a multisystem disease, Behçet disease (BD) has the potential to impact a wide range of organs, including the central nervous system, gastrointestinal tract, eyes, skin, and joints. It is most commonly seen in young adults between 20 and 40 years of age, but it is less frequently diagnosed in children.^[1,2]^

The pathogenesis of BD remains incompletely elucidated; nevertheless, the prevailing hypothesis posits that the disorder stems from an aberrant immunological response precipitated by an exogenous environmental factor within a genetically predisposed host.^[1]^

Although vascular involvement is a major feature of BD, it is not clinically apparent in all patients. Specific complications include large vein thrombosis (1.1%), aneurysms (0.5%), and arterial thrombosis in a smaller percentage (0.154%). Crucially, among these vascular complications, pulmonary aneurysm and major vessel thrombosis are the leading causes of mortality.^[3]^ Here, we present a case of a young man initially presenting with a complex picture of massive hemoptysis and chronic anemia, ultimately managed as BD complicated by extensive thromboembolism involving the pulmonary, caval, and cardiac circulation.

## 2. Case presentation

A 20-year-old male with a known history of rheumatoid disease, a smoking habit, and a history of recurrent oral aphthous ulcers presented with a 2-week history of massive hemoptysis. Associated constitutional symptoms included significant weight and appetite loss, rigors, and a persistent fever of 39°C.

On physical examination, the patient appeared pale. Vital signs were recorded as a blood pressure of 120/40 mm Hg, a pulse rate of 95 beats per minute, a respiratory rate of 20 breaths per minute, and an oxygen saturation of 95%. Respiratory examination revealed diminished breath sounds over the right lung field, while cardiac auscultation and the abdominal and extremity examinations were unremarkable.

Laboratory findings revealed significant anemia, with a hemoglobin level of 7.3 g/dL and a microcytic pattern (mean corpuscular volume of 69 fL), consistent with chronic blood loss. Serological screening was performed to rule out other connective tissue diseases and systemic vasculitides; antinuclear antibodies, anti-double-stranded DNA, and antineutrophil cytoplasmic antibodies (both c-antineutrophil cytoplasmic antibodies and p-antineutrophil cytoplasmic antibodies) were all negative. Antiphospholipid antibodies were also negative, and complement levels (C3, C4) were within normal limits. Koch’s bacillus screening and viral antibodies were negative, but the pathergy test was positive.

**Figure 1.**
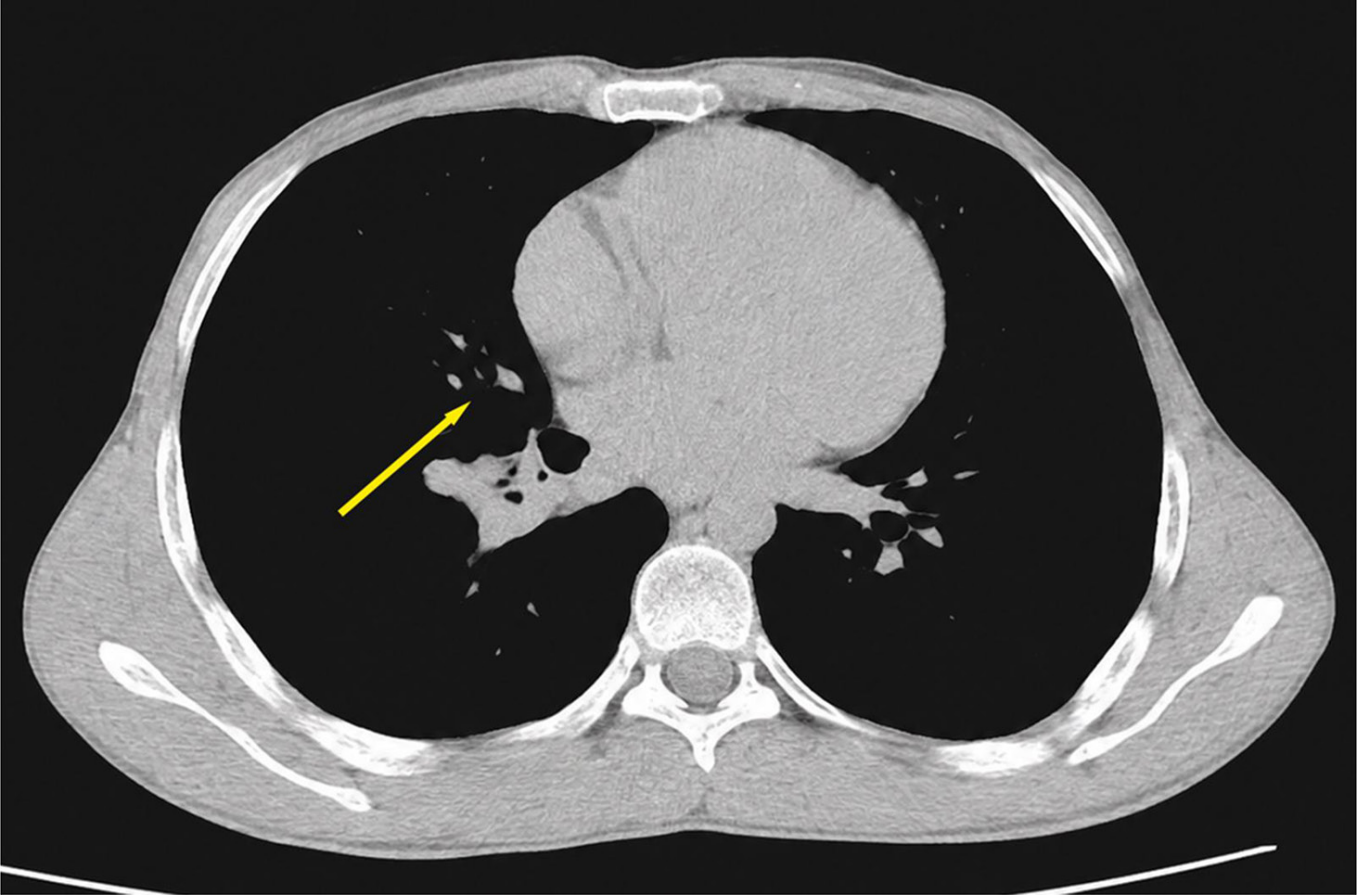
Contrast-enhanced chest CT scan. The images display filling defects within the branches of the right pulmonary artery. CT = computed tomography

**Figure 2.**
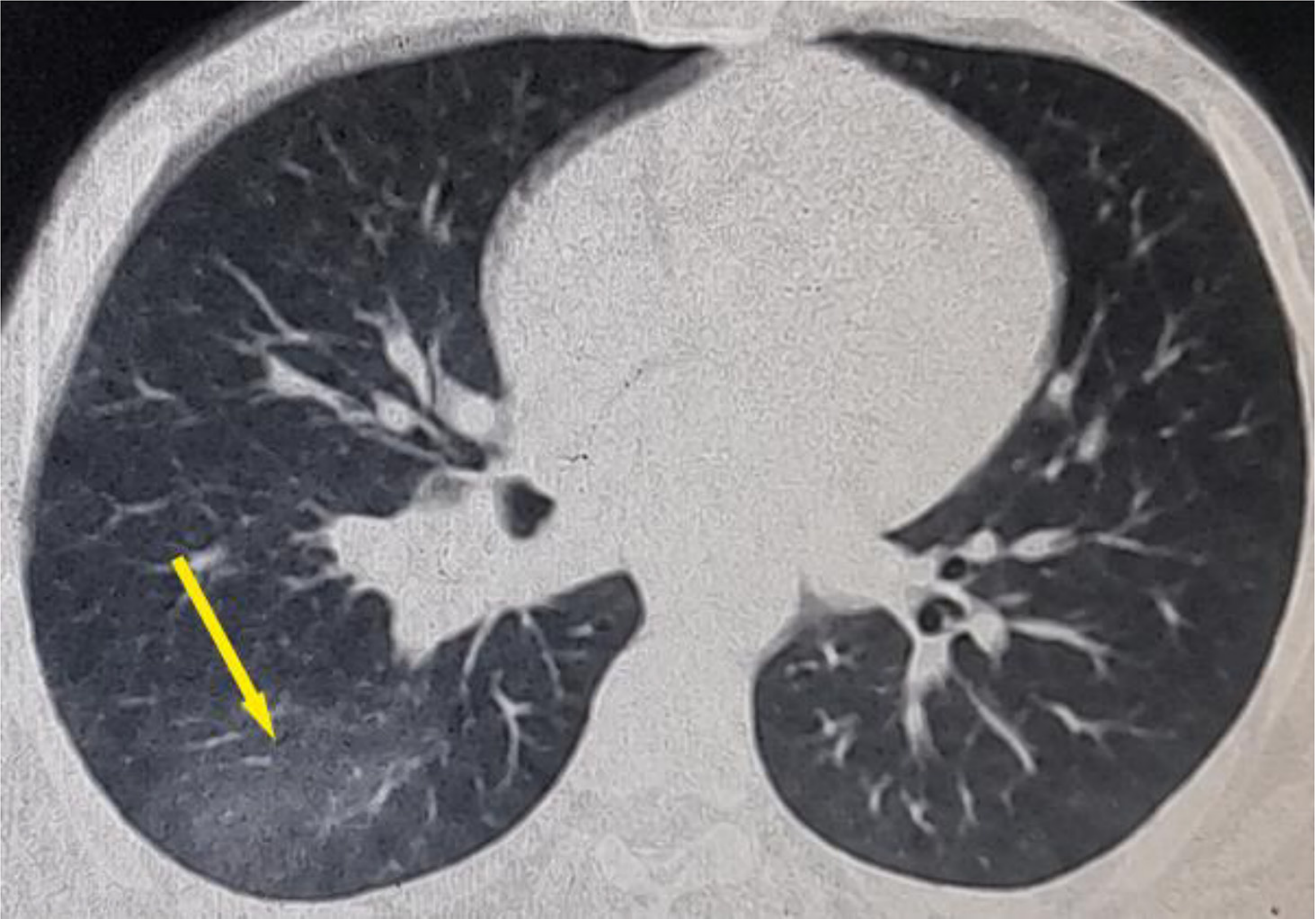
Chest CT scans (lung window). The images display parenchymal abnormalities, including consolidation in the right lung fields. CT = computed tomography.

**Figure 3.**
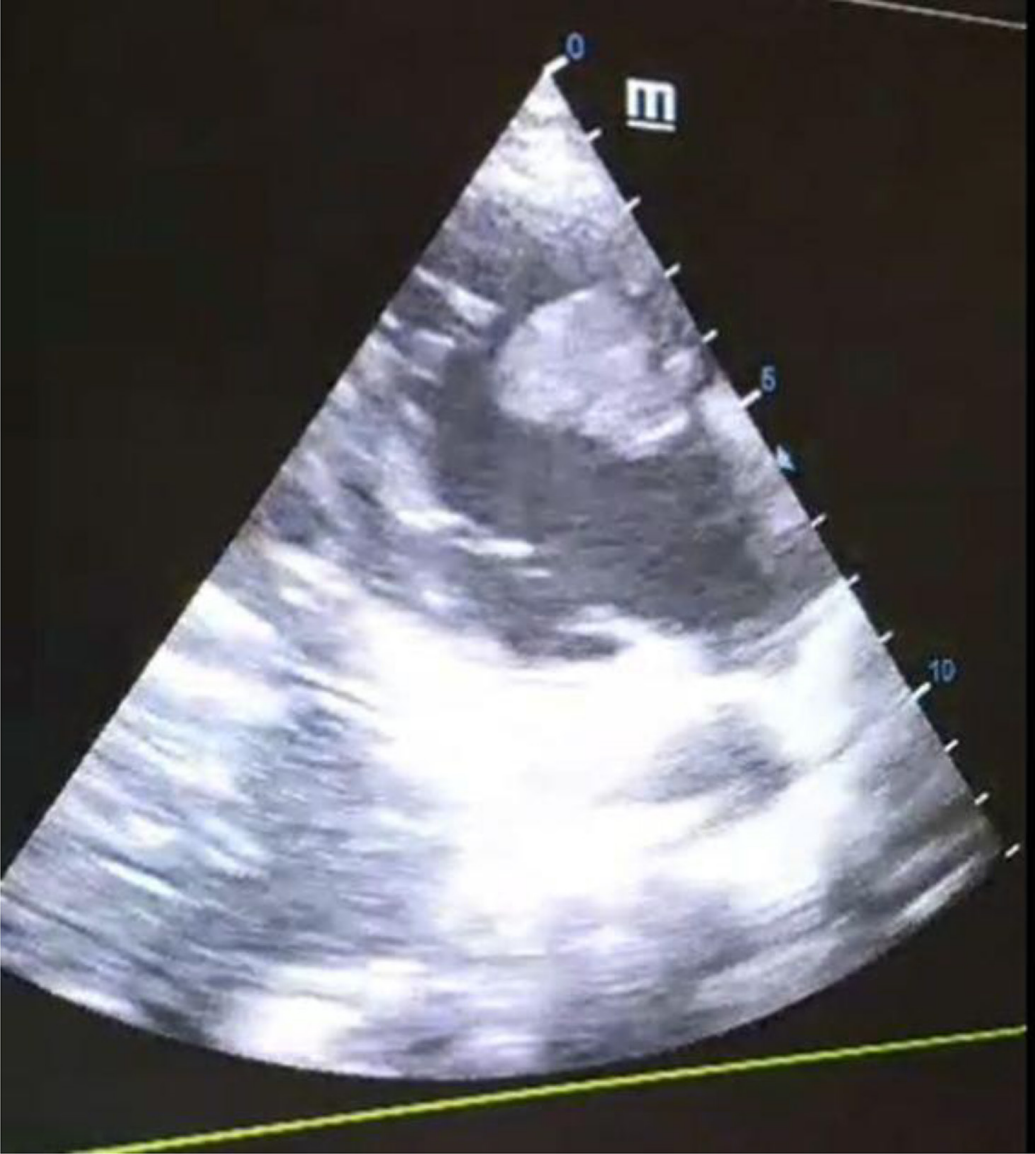
Transthoracic echocardiogram. The image shows a large, well-defined mass (intracardiac thrombus) within the right ventricle.

Imaging studies were particularly revealing. A chest X-ray showed consolidation in the right middle lobe with perihilar congestion. A noncontrast chest computed tomography (CT) scan showed irregular thickening of the bronchi in the right middle and lower lobes, along with multiple subpleural nodules and a basal parenchymal opacity. The contrast-enhanced chest and abdominal CT scan showed extensive thrombosis, including in the inferior vena cava, the right atrium, and multiple pulmonary veins, especially on the right side (Figs. 1 and 2). Transthoracic echocardiogram and color Doppler echocardiogram confirmed the presence of a sessile thrombus on the wall of the right ventricle and a mobile thrombus within it, respectively (Fig. 3).

To manage the massive hemoptysis, the patient was initially stabilized symptomatically before confirming the diagnosis with absolute bed rest and intravenous tranexamic acid (500 mg twice daily). Given the clinical findings, especially the known history of recurrent oral ulcers and the presence of major vessel thrombosis, the patient was managed for a presumptive diagnosis of BD. The therapeutic strategy involved initiating anticoagulant therapy, commencing with therapeutic doses of Heparin (for bridging) followed by warfarin, alongside a course of intravenous methylprednisolone at a dose of 1 g once daily for 3 consecutive days, which was subsequently transitioned upon discharge to oral prednisolone at a maintenance dose of 1 mg/kg/d. In addition, biological immunotherapy was initiated with adalimumab at a loading dose of 160 mg, followed by a maintenance regimen of 40 mg every 2 weeks. Concurrently, the patient was started on mycophenolate mofetil at a dose of 1 g twice daily as an adjunctive immunosuppressive therapy. Subsequent monthly follow-up with cardiac echocardiograms over a 6-month period demonstrated a significant improvement in the size of the thrombus, confirming a positive response to the treatment.

## 3. Discussion

In BD, vascular involvement characteristically presents as aneurysms – primarily in the pulmonary artery – rather than thrombosis, while cardiac involvement remains a rare but life-threatening complication affecting both venous and arterial structures of the heart.^[4–7]^ Our patient case highlights a rare and severe spectrum of thrombotic vascular involvement in BD. The contrast-enhanced chest and abdominal CT scan showed extensive thrombosis, including in the inferior vena cava, the right atrium, and multiple pulmonary veins, especially on the right side. Furthermore, transthoracic and colored Doppler echocardiograms confirmed the presence of a sessile thrombus on the wall of the right ventricle and a mobile one within it. This presentation illustrates a strong vascular propensity for thrombosis in this specific case, in contrast to the formation of arterial aneurysms, which were not observed.

Formal classification of our patient was established using the International Criteria for Behçet Disease scoring system, where the patient accumulated a total score of 4 points based on the recurrent oral ulcers (2 points), major vascular involvement (1 point), and a positive pathergy test (1 point).^[7–9]^ This diagnostic threshold was fulfilled after rigorously excluding mimicking conditions through negative investigation results for tuberculosis and viral antibodies.

Vascular management in BD is rooted in aggressive immunosuppression to control underlying inflammation. While azathioprine or anti tumor necrosis factor-alpha agents are utilized for standard venous disease, severe manifestations – including vena cava thrombosis and arterial aneurysms – mandate corticosteroid therapy combined with cyclophosphamide. Similarly, treating intracardiac thrombosis typically requires a potent triple regimen consisting of high-dose corticosteroids, an immunosuppressant, and anticoagulation.^[7]^ Our therapeutic approach carefully navigated the divergent strategies documented in medical literature for such clinical dilemmas. For instance, Dogan et al ^[10]^ successfully managed a similar case of Behçet syndrome presenting with an intracardiac thrombus by utilizing anticoagulation to mitigate the high-risk of a fatal pulmonary embolism. Conversely, Samrah et al ^[11]^ advocated for withholding anticoagulants entirely in a patient with multisite thrombosis because of the imminent threat of massive, uncontrolled hemoptysis. Bridging these 2 contrasting paradigms, we implemented a hybrid, sequential regimen tailored to our patient unique risk profile. We initiated emergency treatment with bed rest and tranexamic acid for 48 hours to secure immediate hemostasis and safely allow for diagnostic echocardiography, which subsequently confirmed the mobile right ventricular thrombus. Only after ensuring total cessation of the hemoptysis did we introduce systemic anticoagulation (heparin bridging to warfarin), safely augmented by intravenous methylprednisolone at a dose of 1 g once daily for 3 days, which was subsequently transitioned upon discharge to oral prednisolone at a dose of 1 mg/kg/d, and targeted biological therapy was implemented utilizing adalimumab (160 mg loading dose, followed by 40 mg maintenance every 2 weeks), alongside concomitant immunosuppression with mycophenolate mofetil at a dose of 1 g twice daily. This calculated timing successfully dissolved the high-risk cardiac thrombus while completely avoiding recurrent pulmonary hemorrhage. Subsequent monthly follow-up with cardiac echocardiograms over a 6-month period demonstrated a significant improvement in the size of the thrombus, confirming a positive response to the treatment.

## 4. Conclusion

This case highlights the multifaceted nature of BD as a systemic vasculitis with a highly variable clinical expression. The patient aggressive thrombotic phenotype underscores the heterogeneity of the disease, demonstrating that BD can manifest with critical multisite venous and intracardiac thrombosis even in the absence of classic arterial aneurysms. Crucially, managing concurrent massive hemoptysis and high-risk cardiac thrombi requires a carefully timed, sequential therapeutic approach rather than immediate standardized anticoagulation. Initial stabilization of hemorrhage, followed by aggressive immunosuppression and subsequent tailored anticoagulation, offers a safe and effective pathway to eliminate life-threatening thrombi while avoiding recurrent hemorrhage. Continuous clinical monitoring remains essential to evaluate long-term recurrence patterns and optimize maintenance therapy in these rare thrombotic variants.

## Author contributions

**Writing** – **original draft:** Mohammad Obada Alsadi, Areej Alboudy, Wajd Tareq, Reem Akasha, Bashar Quteishat, Mushrega Abdalla.

**Writing** – **review & editing:** Mohammad Obada Alsadi.

**Supervision:** Amr Hodaifa.
